# Barriers to physical activity in older adults in Germany: a cross-sectional study

**DOI:** 10.1186/1479-5868-8-121

**Published:** 2011-11-02

**Authors:** Anna Moschny, Petra Platen, Renate Klaaßen-Mielke, Ulrike Trampisch, Timo Hinrichs

**Affiliations:** 1 Department of Sports Medicine and Sports Nutrition, University of Bochum, Universitätsstraße 150, 44801 Bochum, Germany; 2 Department of Medical Informatics, Biometry and Epidemiology, University of Bochum, Universitätsstraße 150, 44801 Bochum, Germany

**Keywords:** aged, motor activity, exercise, barrier, determinant, chronic disease

## Abstract

**Background:**

Data on barriers to physical activity in older adults in Germany are scarce. The aim of this study was to analyse barriers to physical activity in a cohort of older adults, allowing comparisons between men and women, and age groups.

**Methods:**

1,937 older adults with a median age of 77 (range 72-93) years (53.3% female) took part in the 7-year follow-up telephone interviews of the getABI cohort. Participants who stated that they did not get enough physical activity were surveyed with respect to barriers to physical activity. Barriers were analysed for all respondents, as well as by sex and age group for cases with complete data. Multivariate logistic regression analysis was performed to evaluate differences between sexes and age groups. The level of significance (alpha < 0.05) was adjusted for multiple testing according to Bonferroni (p < .004).

**Results:**

1,607 (83.0%) participants stated that they were sufficiently physically active. 286 participants rated their physical activity as insufficient and responded to questions on barriers to physical activity completely. The three most frequently cited barriers were poor health (57.7%), lack of company (43.0%), and lack of interest (36.7%). Lack of opportunities for sports or leisure activities (30.3% vs. 15.6%), and lack of transport (29.0% vs. 7.1%) were more frequently stated by female respondents than male respondents. These differences between men and women were significant (p = .003; p < .001) after adjustment for respondents' age. Analyses by age groups revealed that poor health was more frequently considered a barrier to physical activity by participants aged 80+ years compared to the younger age group (71.1% vs. 51.5%). This age-dependent difference was significant (p = .002) irrespective of the participants' sex.

**Conclusions:**

The present study provides relevant data on barriers to physical activity in older adults. By revealing appreciable differences between men and women, and age groups, this study has implications for efforts to increase older adults' physical activity. Promotion and intervention strategies should consider the barriers and tailor measures to the specific needs of older adults in order to reduce their constraints to physical activity.

## Background

Middle-aged and older adults consider staying physically and mentally fit to be one of the most important properties in life [1]. Physical activity has the potential to preserve and improve physical and mental health, as well as health-related quality of life, even in previously sedentary and chronically diseased older adults [2-4]. The evidence for the multifaceted benefits of physical activity is compelling. Nevertheless, the physical activity behaviour of most elderly people does not comply with current guidelines [5-7]. A national survey of adults in Germany [8] revealed that 72.8% of older women and 65.3% of older men (age 65+ years) did not reach the recommended amount of at least 2.5 hours per week of moderate-intensity physical activity. Additionally, one out of two respondents (women: 48.2%, men: 52.8%) stated that they did not engage in any sporting activities. These high rates of insufficiently active older adults highlight the need to better understand the reasons for sedentary behaviour in this population. Containing well-established psychological models of health behaviour change, the Health Action Process Approach by Schwarzer et al. [9] considers barriers to be a relevant element in explaining health-compromising behaviour such as physical inactivity. Supporting this are reviews on correlates of physical activity summarizing that barriers are strongly negatively associated with levels of physical activity [10,11]. Within a Delphi study, 118 experts rated determinants of physical activity. Consensus was reached on perceived barriers to physical activity to be among ten highly relevant factors (of the original 73 items) in predicting the initiation of physical activity [12]. A deeper understanding of barriers to physical activity is thus a necessary prerequisite for developing well-founded promotion and intervention strategies.

While several international studies provide insight on impediments to physical activity [13-27], there is scant knowledge on barriers to physical activity in the elderly population in Germany. In the period from 1992 to 1995, the Bonn study on physical activities in the elderly [28] was the first — and for a long time the only — German survey to investigate barriers to physical activity in insufficiently active older adults. Within the oldest age group of participants aged 70 years and over, well-being without sports (40.0%) was most frequently stated. Over one-third of participants (36.4%) did not exercise for health reasons. Further barriers were: enough other hobbies (34.3%), too exhausting (30.0%), risk of injury too high (28.6%), lack of interest (25.7%), dislike of unfamiliar groups (22.9%), lack of company (20.0%) no knowledge of opportunities (18.6%), attitude that sports are only for younger people (15.7%), no time (13.6%), fear of inability (12.1%), and financial reasons (10.0%). More recently, a second study examined barriers to physical activity in a sample of insufficiently active adults in Germany [29]. In the age group 70+ years, the most frequent barrier (74.7%) was having too few friends to exercise with. 58.4% stated that their health did not allow them to be physically active. 39.0% were inactive due to lack of time, and 14.7% due to lack of opportunities for physical activity in their residential area.

Neither of the two German studies displayed results separately by sex for the age group 70 years and over. Similarly, most international studies presented their results combined for men and women [16,17,19,22-25] or studied only one sex [13,21,30-32]. However, the few studies regarding barriers in middle-aged or older adults by sex revealed significant differences between men and women [14,15,18]. Clark [15] conducted a focus group study among low income adults aged 55-70 years. While women more often discussed psychological and physiological barriers such as perceived abilities, pain and fear of pain, men stated lack of motivation as the primary barrier. The author himself admittedly remarks that the information reported in the paper is incomplete, since "much of the most valuable input was in the form of group interest and agreement" [15]. Booth et al. [14] studied perceived barriers to physical activity among older adults from Australia. While the six most frequently mentioned barriers were the same for men and women, there were some substantial differences with regard to percentages. The same study reported changes in percentages for barriers depending on the age of respondents (60-64, 65-69, and 70+ years). It thus seems reasonable to analyse barriers not only separately by sex but also separately for age groups, though the oldest age group analysed in literature with regard to barriers was predominantly defined as "70 years and over" [14,29,33]. To the authors' knowledge, there only very few international [20] and no German studies exist investigating factors that impede physical activity in adults over the age of 70 years, separately by sex and by age group.

Consequently, the aim of the study was to analyse barriers to physical activity in a cohort of older primary health care patients allowing comparisons between men and women, and between age groups of older adults.

## Methods

### Design and participants

The "German epidemiological trial on ankle brachial index" (getABI) is a prospective observational cohort study. Details of its design and methods have already been published [34,35]. In short, each of the participating 344 general practitioners consecutively recruited on average 20 eligible patients seeking primary health care during a predetermined week in October 2001 and fulfilling the inclusion criteria (age ≥ 65 years, being legally competent and able to cooperate appropriately, and providing written informed consent). The only exclusion criterion was life expectancy ≤ 6 months. A total of 6,880 primary health care patients were included in the study. Within the 7-year follow-up period, 1,302 patients died. The remaining 5,578 patients were contacted by letter and by one telephone call to evaluate their willingness to participate in the computer-assisted telephone interview at the 7-year follow-up. 196 patients were unable to participate in the interview; another 3,445 patients did not participate in the interview for several other reasons (not reachable, did not want to be contacted by telephone, refused to participate in the telephone interview). Finally, 1,937 patients (response rate 34.7%) were available for the computer-assisted telephone interviews at the 7-year follow-up. Comparing these participants to non-participants revealed the following significant differences: participants were younger at baseline (median age (range): 70 (65-85) years vs. 72 (65-91) years), were more often male (46.7% vs. 35.7%), and were better educated (qualification higher than basic secondary school: 40.2% vs. 27.1%). The subjects' median age was 77 (range 72-93) years, 53.3% were female. Results reported in this paper mainly refer to cross-sectional data collected during the 7-year follow-up interviews.

The study was approved by the ethics committees of Heidelberg University and Bochum University (Germany), and was conducted according to the "Good Epidemiological Practice" recommendations issued by the "German Working Group Epidemiology" [36].

### Barriers to physical activity

To ensure appropriateness of questions on barriers, subjects were initially asked: "From your point of view, are you sufficiently physically active?" Participants answering "yes" were not queried about barriers. Those who stated that they were not sufficiently physically active were asked for reasons hindering them. They were asked to answer whether they "agree", "partly agree", or "disagree" with the following statements representing frequently reported barriers in older adults [13,14,16,17,19,22-25,28,29] (Table 1):

**Table 1:** Statements on barriers to physical activity and abbreviated designation for use in figures/tables

	Item	Abbreviated designation
1	I haven't any time for physical activity.	Lack of time
2	I am afraid that I will fall or hurt myself during exercise.	Afraid of fall/injury
3	I don't have any company. I would be more active with a partner or in a group.	Lack of company
4	For health reasons, I don't feel I can be more active.	Poor health
5	There are no appropriate sports programmes or leisure facilities for me.	Lack of opportunities
6	I do not have transport to sports programmes or leisure facilities.	Lack of transport
7	I am not interested in physical activity.	Lack of interest

### Descriptive variables

The variables mentioned below were assessed at baseline or during the follow-up telephone interviews of the getABI cohort. They were used to describe participants.

#### Sociodemographic variables

At baseline, the general practitioner documented the participants' sex, date of birth and education level (no qualification - completed basic secondary school — vocational school — university entrance qualification). The participants' native country was assessed during the 5-year follow-up, and the number of persons living in the same household was elicited during the 7-year follow-up telephone interviews.

#### Cardiovascular risk factors

The current smoking status was documented at baseline (smoking no/yes). The participants' waist circumference was measured by study personnel in the general practitioner's practice by standard protocol at the 5- and the 7-year follow-up. Waist circumference at 7-year follow-up was used for analysis. If values were missing, waist circumference at 5-year follow-up was used.

All of the following variables were assessed during the 7-year follow-up telephone interviews.

#### Chronic conditions and number of medications

Participants were asked whether they had one of the following chronic diseases (no/yes): arterial hypertension, coronary heart disease, myocardial infarction, chronic heart failure, diabetes mellitus, peripheral arterial disease, chronic obstructive pulmonary disease, arthritis (degenerative or rheumatoid), osteoporosis. The number of medications was counted based on the participants' specification of drug codes.

#### Walking ability, falls and pain

Walking ability was appraised by asking participants for the need of a walking aid (no aid — cane — rollator — wheelchair-bound — bed-ridden). Falls were defined as "an unexpected event in which the participants come to rest on the ground, floor, or lower level" [37]. Respondents stated falls within the previous 12 months (no/yes), and pain within the previous 3 months (no/yes).

### Statistical analysis

Twenty variables were considered for describing patient characteristics (each no/yes, if not indicated otherwise): sex (female/male), age (< 75/75-79/80-84/≥85 years), country of origin (foreign country/Germany), qualification (no qualification or completed basic secondary school/vocational school/university entrance qualification), currently smoking at baseline, waist circumference (women </≥88 cm; men </≥102 cm) [38], arterial hypertension, coronary heart disease and/or myocardial infarction, chronic heart failure, diabetes mellitus, peripheral arterial disease, chronic obstructive pulmonary disease, arthritis, osteoporosis, number of chronic diseases (0-1/2-3/4-5/≥6), number of medications (0-2/3-5/6-8/9-11/≥12), need for a walking aid, falls within the preceding 12 months, pain during the preceding 3 months. The univariate distribution of these variables is represented by absolute frequencies and percentages.

The evaluation of barriers to physical activity was done by summarizing participants who agreed or partly agreed to each statement. Barriers were analysed for all respondents as well as by sex and by age group (</≥80 years). Multivariate logistic regression analysis was performed to evaluate differences between sexes adjusted for age group and vice versa. The level of significance (α = 0.05) was adjusted for multiple testing according to Bonferroni (p < .004). Barriers to physical activity in men and women by age group were analysed descriptively. PASW Statistics 18 (version 18.0.2) was used for statistical analysis.

## Results

### Participants

**Figure 1:**
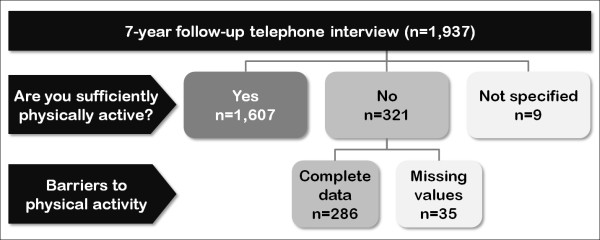
**Sequence of questions and formation of three subgroups: 1) subjectively sufficiently active patients (n = 1,607); 2) subjectively insufficiently active patients with complete data on barriers (n = 286); 3) patients with incomplete data (n = 44)**.

Of the 1,937 participants, 1,607 (83.0%) stated that they were sufficiently physically active. 321 (16.6%) subjects said they were not sufficiently physically active. These were queried about barriers to physical activity. Figure 1 illustrates the sequence of questions and the formation of different subgroups.

The focus is on 286 participants who rated themselves as insufficiently physically active and responded to the statements on barriers to physical activity completely. A total of 44 participants were excluded from analyses due to incomplete data.

Characteristics of the three subgroups are presented in Table 2. Data was missing significantly more frequently for subjects in need of a walking aid. Among participants to be analysed with regard to barriers, 71.1% exhibited a waist circumference above reference values, 75.2% had at least two chronic diseases, 65.0% had had pain within the preceding 3 months, and 63.6% took more than 5 medications. These percentages were about 10-15% lower in participants who stated that they were sufficiently physically active.

**Table 2:** Characteristics of getABI patients at time of the 7-year follow-up by subgroups

	Subjectively insufficiently active patients + complete data on barriers (n = 286)		Subjectively sufficiently active patients (n = 1607)			Patients with incomplete data (n = 44)		
	
	n	%	n	%	chi^2 ^	n	%	chi^2 ^
**Sociodemographic variables**								
Male	**141**	**49.3**	741	46.1	n.s.	22	50.0	n.s.
Age (years)								
< 75	**83**	**29.0**	359	22.3	n.s.	7	15.9	n.s.
75-79	**113**	**39.5**	692	43.1		20	45.5	
80-84	**64**	**22.4**	410	25.5		10	22.7	
≥ 85	**26**	**9.1**	146	9.1		7	15.9	
Native country Germany	**258**	**90.2**	1470	91.5	n.s.	37	84.1	n.s.
Qualification								
No qualification or basic secondary school	**148**	**52.9**	963	60.6	n.s.	24	55.8	n.s.
Vocational school	**79**	**28.2**	379	23.8		13	30.2	
University entrance qualification	**53**	**18.9**	248	15.6		3	14.0	
Living alone	**93**	**32.5**	594	37.0	n.s.	13	29.5	n.s.

**Cardiovascular risk factors**								
Currently smoking (baseline)	**24**	**8.4**	102	6.3	n.s.	1	2.3	n.s.
Waist circumference^#^: women ≥ 88 cm; men ≥ 102 cm	**197**	**71.1**	966	61.8	*	31	73.8	n.s.

**Medical conditions and medication**								
Hypertension	**199**	**69.6**	1018	63.4	*	30	68.2	n.s.
Coronary heart disease and/or myocardial infarction	**82**	**28.7**	400	24.9	n.s.	16	36.4	n.s.
Chronic heart failure	**75**	**26.2**	284	17.7	*	11	25.0	n.s.
Diabetes mellitus	**105**	**36.7**	360	22.4	*	14	31.8	n.s.
Peripheral arterial disease	**45**	**15.7**	175	10.9	n.s.	9	20.5	n.s.
Chronic obstructive pulmonary disease	**45**	**15.7**	187	11.7	n.s.	6	13.6	n.s.
Arthritis (degenerative or rheumatoid)	**134**	**46.9**	535	33.3	*	19	43.2	n.s.
Osteoporosis	**48**	**16.8**	221	13.8	n.s.	5	11.4	n.s.
Number of chronic diseases^†^								
0-1	**71**	**24.8**	631	39.3	*	12	27.3	n.s.
2-3	**141**	**49.3**	742	46.2		22	50.0	
4-5	**66**	**23.1**	205	12.8		9	20.5	
≥ 6	**8**	**2.8**	27	1.7		1	2.3	
Number of medications								
0-2	**22**	**7.7**	220	13.7	*	2	4.5	n.s.
3-5	**82**	**28.7**	559	34.8		10	22.7	
6-8	**103**	**36.0**	530	33.0		16	36.4	
9-11	**57**	**19.9**	212	13.2		9	20.5	
≥ 12	**22**	**7.7**	85	5.3		7	15.9	

**Need for walking aid**	**68**	**23.8**	243	15.1	*	16	39.0	*

**Falls (preceding 12 months)**	**78**	**27.3**	340	21.2	*	10	22.7	n.s.

**Pain (preceding 3 months)**	**186**	**65.0**	815	50.7	*	30	68.2	n.s.

^# ^Waist circumference at 7-year follow-up was used for analysis. If this value was missing, waist circumference at 5-year follow-up was used.

^† ^referring to the chronic diseases listed above

chi^2 ^Pearson's chi-square test (p < .05)

n.s. No significant difference in comparison to the group of subjectively insufficiently active patients

* Significant difference in comparison to the group of subjectively insufficiently active patients

### Barriers to physical activity

**Figure 2:**
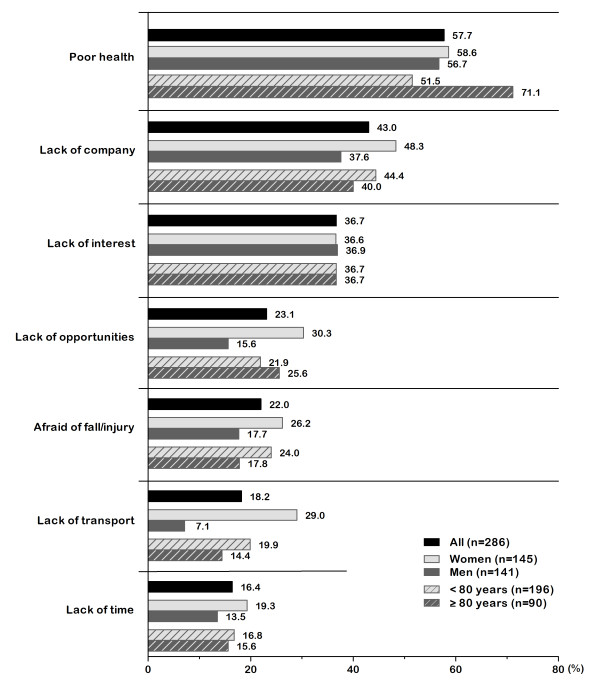
**Barriers to physical activity in subjectively insufficiently active older adults**.

Barriers are presented in descending order of frequency for the sample of subjectively insufficiently active getABI patients (Figure 2). At a percentage of 57.7%, poor health ranked first as a barrier to physical activity. A lack of company applied to 43.0% of participants, and over one-third of respondents were not interested in physical activity. Lack of time was the least important barrier and impeded physical activity in 16.4% of respondents.

Figure 2 additionally shows percentages by sex and by age group. There were negligible differences between men and women in ratings with regard to the barriers poor health and lack of interest. The most distinct differences between men and women concerned the barriers lack of opportunities and lack of transport. The belief that there are no appropriate opportunities for sports or leisure activities was a barrier for 15.6% of men, while the percentage of female participants was twice as high (30.3%). Lack of transport applied to 29.0% of women, whereas it was least important (7.1%) for male respondents. Multivariate logistic regression analyses revealed that these sex-related differences were significant (lack of opportunities: p = .003; lack of transport: p < .001), irrespective of participants' age (see Table 3). When examining barriers by age group, there was an increase from 51.5% in the younger age group to 71.1% in those aged 80 years and over referring to the barrier poor health. In multivariate logistic regression analyses, this was the only barrier that was considered significantly more frequently in participants aged 80 years and over (p = .002), independent of sex (see Table 3). Additional descriptive information regarding percentages for men and for women by age group is presented in Table 4. Regarding the barrier poor health, the percentage was 10.1% higher in the age group of women, and 28.8% higher in the older age group of men compared to their younger counterparts, respectively.

**Table 3:** Association between both gender and age group and barriers to physical activity (multivariate logistic regression models; n = 286)

	Gender (reference: men)		Age group (reference: age < 80 years)	
	
	Adjusted odds ratio^† ^[99.63% confidence interval]	p-value	Adjusted odds ratio^# ^[99.63% confidence interval]	p-value
**Poor health**	1.14 [0.56;2.33]	0.582	2.34 [1.06;5.19]	0.002*
**Lack of company**	1.54 [0.76;3.09]	0.075	0.86 [0.40;1.84]	0.566
**Lack of interest**	0.99 [0.48;2.01]	0.953	1.00 [0.46;2.15]	0.988
**Lack of opportunities**	2.41 [1.02;5.68]	0.003*	1.31 [0.55;3.17]	0.368
**Afraid of fall/injury**	1.62 [0.69;3.76]	0.099	0.71 [0.28;1.82]	0.287
**Lack of transport**	5.26 [1.77;15.69]	< 0.001*	0.74 [0.26;2.13]	0.411
**Lack of time**	1.53 [0.60;3.93]	0.190	0.94 [0.34;2.59]	0.857

* significant according to the adjusted level of significance p < .004

^† ^adjusted for age group

^# ^adjusted for gender

**Table 4:** Barriers to physical activity in subjectively insufficiently active women and men by age group

	Women		Men	
	
	**< 80 yrs**. (n = 104)	**≥ 80 yrs**. (n = 41)	**< 80 yrs**. (n = 92)	**≥ 80 yrs**. (n = 49)
**Poor health**	55.8	65.9	46.7	75.5
**Lack of company**	51.0	41.5	37.0	38.8
**Lack of interest**	36.5	36.6	37.0	36.7
**Lack of opportunities**	29.8	31.7	13.0	20.4
**Afraid of fall/injury**	27.9	22.0	19.6	14.3
**Lack of transport**	31.7	22.0	6.5	8.2
**Lack of time**	19.2	19.5	14.1	12.2

## Discussion

This study investigated barriers to physical activity in a community sample of older primary health care patients in Germany. To the authors' knowledge, this is the first German study to report barriers to physical activity separately for older men and women, and for different age groups of older adults.

*Poor health *emerged as the most important barrier to sufficient physical activity in participants in the 7-year follow-up telephone interviews. Similarly, it was a frequently cited barrier in different cohorts of older adults in research conducted in Germany and internationally [13-16,18,20,23-25,27,29,32,33,39]. Moreover, there is convincing evidence from epidemiology that poor self-rated health and low perceived physical abilities are in fact strongly associated with lower physical activity among older adults [10,40,41]. Poor health was an equally relevant barrier for men and women of the getABI cohort. Results in the literature regarding gender-related differences are conflicting [14,18,20,33]. In contrast, the growing importance of health as perceived barrier to physical activity with increasing age is consistently reported in other cross-sectional research [14,33] and is confirmed by a longitudinal survey of older adults in Finland [20]. The present study even highlights the sharp increase in percentages from the younger old to those aged 80+ years, independent of participants' sex. Qualitative studies add insights regarding health as a barrier to physical activity. During discussion of health-related problems that hamper physical activity, participants specified heart problems, arthritis, knee or back problems, or functional limitations as barriers [25,27]. However, these conditions do not constitute contraindications to physical activity. In contrast, given the overwhelming evidence, physical activity is strongly recommended for older adults with chronic diseases and functional limitations [3]. They would greatly profit from individually adapted regular physical activity and exercise in terms of preventing progress of disease and disability, and preserving autonomy and health-related quality of life.

Negative outcome expectations concerning physical activity in old age may partly explain why so many older adults refrain from increasing their physical activity level when their health is already compromised [10,25,42]. Many older adults are afraid of "overdoing it", and fear chest pain, injury or falling [15,23,25]. Within the getABI cohort, *fear of injury or falling *was stated as a barrier by 22.0% of participants. This is generally in line with other quantitative studies in which percentages ranged from 23.8%-28.6% [19,33]. Community-dwelling adults aged 75-81 years with moderate or severe mobility limitation more frequently stated poor health, fear of falling or injury, and negative experiences to be barriers to physical activity [26]. Fear may result from a lack of experience with physical activity, as well as from negative experiences: "I used to walk around the building, but I fell once and hurt myself" [23]. Although the benefits of physical activity far outweigh the risks for almost everyone [43], the individual concerns have to be taken seriously. Health care professionals and other providers bear responsibility to minimize negative and facilitate positive experiences with regard to physical activity.

*Lack of company *was a barrier for 43.0% of participants. Denk reported a rate of 20% [33], whereas Rütten et al. found that "having few friends to exercise with" (74.7%) was the barrier most frequently stated in the age group 70 years and over [29]. Several international studies confirm the relevance of company for physical activity behaviour in older adults [19,24,39]. While one study found comparable percentages for men and women [14], another one reported higher rates for female (30.1%) than male (12.4%) respondents [18]. Accordingly, our own data indicate that having no company was more relevant for women in the getABI cohort. Lack of supportive and motivating companionship is certainly one relevant aspect in this context. Beyond that, the lack of company may play a key role in aged men and women who are concerned about their safety when increasing physical activity levels. Focus group discussions highlight this issue. One participant stated, "Well, I find that I am afraid of falling and being alone" [25]. Another one wondered what could happen if he or she decided to exercise: "Since I am alone, I could be lying there for days" [23]. With a view to the promotion of physical activity, walking is the older adults' preferred activity [44]. At the same time, it is considered to be an ideal activity for this population because of low structural barriers: it does not require special equipment, clothing or venue, is for free, and can be done alone [18]. However, the lack of company, and concomitant concerns about safety have to be considered, because they may thwart physical activity promotion efforts.

*Lack of transport *to sports programmes and facilities, and *lack of opportunities *were relevant barriers for getABI patients. Lack of opportunities was also found to be a barrier in the two German studies [29,33] and in international research [19,22,24]. It remains unclear whether this lack of sports programmes and facilities is real or perceived. Studying older adults' wishes and needs with regard to sports programmes and examining local opportunities would help to elucidate this issue. Balancing possibilities for advising older adults on existing opportunities, Schofield et al. [45] report that the general practitioner is the most trusted source of physical activity information, especially among older adults and those with multiple chronic diseases. Building networks between general practitioners and local providers may simplify briefing of patients about opportunities in their vicinity and raise their awareness of available sports programmes which could help them increase their physical activity and manage their health [46].

Perceived access to facilities was judged to be another relevant determinant for the initiation of physical activity in older adults [42]. Several studies found transport difficulties to be a barrier to physical activity [24,25,30,39]. Daily access to a car and lack of transport were shown to be associated with participation in leisure time physical activity in a sample of 65-84-year-old men and women [17]. Inadequate availability, frequency and reliability of affordable transport were discussed among participants of a focus group [39]. Moreover, distance to sports facilities emerged as a barrier to participation in fitness programmes for managed Medicare enrolees aged 65 years and over. Participants lived nearer to facilities than non-participants. Furthermore, among participants distance was correlated with frequency of participation in the unstructured fitness programme [47]. Generally, facilities that are not within walking distance necessitate transportation. However, in the course of aging, individuals possessing a driving licence may lose their ability to drive or may feel increasingly unsafe in road traffic, both resulting in a reduction or cessation of driving a car. Using public transport is usually more time-consuming. Additionally, it may evoke uncertainty and be especially exhausting in mobility restricted older adults. Both lack of transportation and lack of appropriate opportunities were significantly more prevalent in female getABI patients than their male counterparts in the same cohort, irrespective of respondents' age. This finding deserves further study, although support from literature is lacking. A study of adults aged 60+ years [14] did not find gender-related differences with regard to the barrier "There's no suitable facility nearby". No study reported lack of transport as barrier to physical activity broken down by sex. Reasons for our own findings are unclear. However, the fact that 75.9% of men but only 26.6% of women starting from the age of 75 have a driving licence [48] may partly explain gender-related differences regarding transportation as a barrier to physical activity. However, the high rates of women reporting these barriers in the present survey may partly explain lower sports participation rates observed in women compared to men [49].

*Lack of time *was a rather infrequent barrier to physical activity in getABI patients. The relevance of this barrier seems to decrease in the course of life [20]. Booth et al. [14] reported decreasing percentages with increasing age of older adults (age group 60-64 (27.3%), 65-69 (16.1%), 70+ (7.1%)). No differences could be found between getABI patients between the ages of 72 and 79 and aged 80+ years with regard to time as a barrier to physical activity. All these findings are plausible given the entry into retirement age, the cessation of work-related time commitments, and the restructuring of leisure time in older adults. Nevertheless, a review on determinants of physical activity in older adults points out that lack of time is weakly, yet negatively associated with overall physical activity [10]. It may be assumed that in a population of aged adults, lack of time is not only a matter of new time commitments, but also a question of priorities for leisure time activities and a lack of interest in physical activity. A study in a sample of middle-aged and older women compared time commitments and perceived lack of time for physical activity [50]. Besides time spent in work, household or "family responsibilities", women spent 28 hours per week in sedentary leisure-time activities. A comparable dimension may be assumed for aged men as well. In the present study, a *lack of interest *in physical activity was stated by over one-third of participants, without difference between men and women, or age groups. Percentages in literature range from 10.2% to 45.9% [19,20,22,33]. A study in a sample of 409 men and women aged 65-84 years revealed that those who lacked interest in physical activity often or daily had 7.8 times higher odds (95% confidence interval: 2.68-22.58, adjusted for covariables) of being physically inactive in their leisure time [17]. Consequently, strategies for integrating health-enhancing physical activity into everyday life of people in old age have to consider existing time commitments, as well as older adults' leisure time priorities and interests. Efforts to raise interest in physical activity should highlight benefits of physical activity, not only for health but especially those relating to socializing, enjoyment, relaxation and physical and mental well-being.

### Limitations of the study

The subjective rating of being sufficiently physically active is a limitation of the present study. Participants' physical activity level was not objectively measured. Furthermore, it is unclear how getABI patients interpret the term "sufficient". We agree with Booth et al. [14], who implied the same subjective rating and remarked that "[...] a better understanding of the beliefs of older adults with regard to [...] the amount and type of activity required for health would be generally informative and would help resolve this issue". Regarding the assessment of barriers, there was no standardized, German tool at the time of the 7-year follow-up. However, asking about barriers is deemed to be closely related to people's subjective justifications for physical inactivity [51]. Hence, this study asked about reasons of insufficient physical activity, and considered barriers frequently reported in research undertaken in Germany and internationally.

The response rate in the 7-year follow-up telephone interview was 34.7%. As expected, participants were younger and better educated compared to non-participants. Furthermore, it can be assumed that the willingness and ability to continue to participate in a longitudinal trial after 7 years is higher in healthier persons. Participants who had moved to a nursing home during the follow-up period were no longer able to participate. Therefore, the results of the present study apply to relatively fit seniors who are still able to visit their general practitioner and participate in a telephone interview. The probable selection towards the healthier patients from baseline to the 7-year follow-up in the getABI cohort may have influenced the results. For example, the percentages of persons reporting poor health or fear of falling as barriers to physical activity may have been underestimated.

## Conclusions

The present study provides relevant data on barriers to physical activity in older adults. By revealing appreciable differences between men and women, and age groups, this study has specific implications for efforts to increase older adults' physical activity level. Promotion and intervention strategies should consider the barriers and tailor measures to the specific needs in order to reduce older adults' constraints to physical activity.

## Competing interests

The authors declare that they have no competing interests.

## Authors' contributions

TH and PP obtained the research grant for the project "Physical activity, multimorbidity and polypharmacy in the elderly" within the PRISCUS research cooperation [52] and initiated the specific data collection in the getABI cohort. TH coordinated the project. AM and TH conceived the research question and the statistical design of the present study. AM edited the data and performed the statistical analyses. RKM participated in data preparation and double-checked the statistical analyses. AM, TH and UT interpreted the data. AM drafted the manuscript. All authors revised the manuscript critically for important intellectual content. All authors approved the version to be published.
